# Integrating the Population Perspective into Health System Performance Assessment (IPHA): Study Protocol for a Cross-Sectional Study in Germany Linking Survey and Claims Data of Statutorily and Privately Insured

**DOI:** 10.15171/ijhpm.2019.141

**Published:** 2020-01-20

**Authors:** Miriam Blümel, Julia Röttger, Julia Köppen, Katharina Achstetter, Reinhard Busse

**Affiliations:** Department of Health Care Management, Berlin Centre for Health Economics Research, Technische Universität Berlin, Berlin, Germany.

**Keywords:** Health System Performance Assessment, Population Perspective, Germany, Data Linkage, Claims Data

## Abstract

**Background:**Health system performance assessment (HSPA) is a major tool for evidence-based governance in health systems and patient/population-orientation is increasingly considered as an important aspect. The IPHA study aims (1) to undertake a comprehensive performance assessment of the German health system from a population perspective based on the intermediate and final dimensions defined by the World Health Organization (WHO) and (2) to identify differences in HSPA between (a) common user characteristics and (b) user types, which differ in their interactions and patterns of action within the health system.

**Methods and Analysis:** A cross-sectional survey was conducted between October and December 2018 with statutorily and privately health insured to assess the German health system from a population perspective related to the past 12 months. The random sample consists of 32 000 persons insured by AOK Nordost and 20 000 persons insured by Debeka. Data from the survey will subsequently be linked with health insurance claims data at the individual level for each respondent who has given consent for data linkage. Claims data covers the time period January 1, 2017 to June 30, 2018. The combination of the 2 data sources allows to identify associations between insured patient characteristics and differences in the assessment of health system performance. The survey consists of 71 items measuring all final and intermediate health system goals defined by the WHO and user characteristics like health literacy, self-efficacy, the attention an individual pays to his or her health or disease, the personal network, autonomy, compliance and sociodemographics. The claims data contains information on morbidity, care delivery, service utilization, (co)payments and sociodemography.

**Discussion:** The study represents a promising attempt to perform a holistic HSPA using a population perspective. For this purpose, a questionnaire was designed that contains both validated and new items in order to collect data on all relevant health system dimensions. In particular, linking survey data on HSPA with claims data is of high potential for assessing and analysing determinants of health system performance from the population perspective.

## Background

Health system performance assessment (HSPA) is increasingly becoming a major tool for evidence-based governance in modern health systems. HSPA is defined as the process of monitoring, evaluating, communicating and reviewing the extent to which different aspects of a healthcare system can achieve previously defined goals.^1^ Several countries and international organizations, eg, Organisation for Economic Co-operation and Development (OECD) and the World Health Organization (WHO), have implemented initiatives and programs to investigate the performance of single sectors or the overall health system based on different frameworks.^2-4^

**Figure 1:**
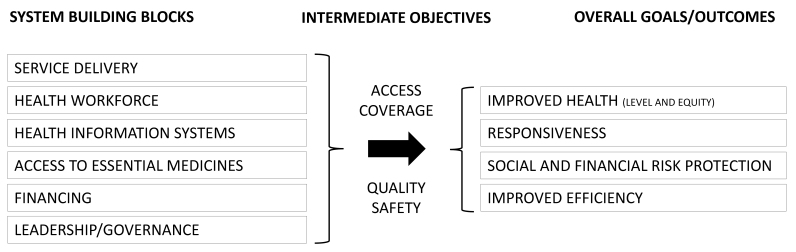
Framework for an HSPA.^5^ Abbreviation: HSPA, Health System Performance Assessment.

In 2000, the WHO introduced its Framework for HSPA to measure the performance of health systems, which has been modified and refined over subsequent years. As shown in Figure 1, the Framework takes into account both the ultimate goals of a health system, such as population health, social and financial risk protection, efficiency, health system responsiveness and inequality in healthcare, and intermediate objectives, ie, access, coverage, quality and safety.^5,6^

The intermediate objectives *access* and *coverage* can be viewed in terms of the ease with which the population can access healthcare services. This, in turn, comprises the following 4 dimensions: (*i*) population coverage, (*ii*) service coverage (ie, services included in the benefit basket), (*iii*) cost coverage, ie, affordability/cost sharing, and (*iv*) availability of services (with possible access barriers being distance, waiting times, and lack of choice of providers).^7^

The 2 intermediate objectives *quality* and *safety* relate to patients’ interactions with the healthcare system and services they receive. Quality refers to the appropriateness of treatment (eg, receiving the correct treatment). Another aspect of quality is the availability of data on quality indicators (transparency of quality, public reporting), which can be subsumed in this dimension. In contrast to quality, safety captures critical incidents, (unexpected) adverse outcomes and negative health effects stemming from the process of care.^8^

These intermediate objectives have a direct impact on overall goals or outcomes within a health system and should therefore be part of an HSPA. All 4 intermediate objectives influence the health of the population overall and the distribution of health across different population groups (eg, by region, socio-demographic or socio-economic aspects), both of which are captured in the overall outcome *improved health*.

*Social and financial risk protection* is especially influenced by the intermediate objective of *coverage*. Financial risk protection within a health system is determined by how funds are raised and how they are pooled to spread risk across population groups.^7^ This dimension covers the extent to which people are financially protected from the consequences of ill health.

*Improved efficiency* was added as an explicit goal in the 2007 version of the WHO framework. Efficiency captures the relationship between input and outputs/outcomes of a health system. Exemplary efficiency measures are “costs per hospital stay” (as an indicator of technical efficiency) or, in order to assess the efficiency of a system, costs spend in relation to final goals (eg, population health measured by attributable mortality). Additionally, several approaches and indicators are currently under discussion or development as ways to assess efficiency in the delivery of healthcare, eg, by measuring the use of antibiotics or identifying duplicate tests.^9,10^

By defining health system *responsiveness* as an outcome of successful health systems, the WHO explicitly incorporated the population’s view within their performance framework. Health system responsiveness comprises the categories “client orientation” and “respect for person” and captures the population’s legitimate expectations of their interactions with the health system.^11,12^ This outcome relates to the whole population as it captures all interactions with the health system and is not restricted to services received.

Patient/population-orientation is increasingly considered an important aspect of health systems and thus also of the assessment of health system performance.^13^ It is therefore not surprising that there are several other approaches to include the patient perspective in the assessment of (aspects of) health system performance, such as the patient-reported outcome measures (connected to the WHO dimensions *quality* and *improved health*)^14^ or patient-reported experience measures (connecting to the WHO dimension *responsiveness*).^15^ Nevertheless, no attempts have been made to incorporate the population perspective within HSPA in a systematic fashion covering all intermediate objectives and overall goals of the WHO framework.

Another aspect that has received little consideration in HSPA research to date is the observation that different attitudes and behaviours of users of a health system may strongly influence how the health system is assessed. While socio-economic, health and regional differences have already been examined in this regard,^16^ differences in the expectations that individuals have of their health system have not yet been subject to HSPA research. These differences may either be subjectively reported or objectively identifiable in performance for specific population groups.^17^

The proposed study, entitled “Integrating the Population Perspective into Health System Performance Assessment” (IPHA), seeks to close this gap in the evidence. The study aims (1) to undertake a comprehensive performance assessment of the German health system from a population perspective based on the intermediate and final dimensions defined by the WHO and (2) to identify differences in HSPA between (*a*) common user characteristics (eg, socio-economic status, regional variation, morbidity) and (*b*) user types, which differ in their interactions and patterns of action within the health system. This study protocol explains the study methods, development of the survey instrument and the linkage of data in detail.

## Methods/Design

The study draws upon data from 2 sources. To measure health system performance from a population perspective, a cross-sectional survey will be conducted. Data from the survey will subsequently be linked with health insurance claims data at the individual level for each respondent. Linking survey and claims data in this manner will allow the subjective assessments and attitudes of the respondents to be combined with objective data on morbidity and the use of healthcare services.

### Sample

The target sample of this study is the German population. However, due to the planned linkage of survey data and claims data, it is not possible to draw a population sample. Statutory health insurance (SHI) consists of 110 individual sickness funds that cover around 88% of the population, while 41 private health insurance (PHI) companies cover another 11%.^18-20^ The large number of sickness funds and PHI companies in Germany makes the associated organisational and data protection requirements impracticable. However, in order to create a representative image of the population, both a random sample of SHI insured persons from one large German sickness fund (AOK Nordost – covering 1.8 million people in the states Berlin, Brandenburg and Mecklenburg-West Pomerania and a random sample of persons with full substitutive PHI from a private insurance company (Debeka – covering 2.4 million people nationwide) are drawn. The sampling includes all persons ≥18 years who are insured in the AOK Nordost and Debeka, respectively, from at least January 1, 2015 until the date of the sampling. Insured who had no contact with the healthcare system during the observation period are also included in the sample. Exclusion criteria are (1) beneficiaries of long-term care degree of 4 or 5, (2) persons with considerably reduced everyday abilities, and (3) stay in a hospice. In addition, only insured persons who have not already participated in another survey by the respective health insurance within the last 2 years will be contacted. Due to the structure of the insured of the respective health insurance, the sample of privately insured persons was stratified by age, gender, and eligibility to aid allowances, and the sample of SHI insured was stratified by age. The total sample consists of 32 000 persons insured by AOK Nordost and 20 000 persons insured by Debeka. The sample size is based on (1) the experience with the response rate of a previous study in which survey data was also linked with SHI claims data^21^ and (2) on the assumption that privately insured people have a lower response rate due to their age structure and occupational status.

### Survey Data

The survey was conducted between October and December 2018 and included several questions relating to the last 12 months. Each person in the final survey sample receives a mailing including a cover letter, a study description, the questionnaire, the declaration of consent, and a stamped return envelope. If participants so choose, they may complete the questionnaire online using a link provided in the cover letter to the paper questionnaire. The questionnaire was written in German language only.

The questionnaire contains a total of 72 items, of which 33 are related to the intermediate and final outcomes of the HSPA framework. These will be used as dependent variables for the subsequent analyses. While there are many validated items for some dimensions, such as health and access, there are very few for others, such as efficiency.

**Figure 2:**
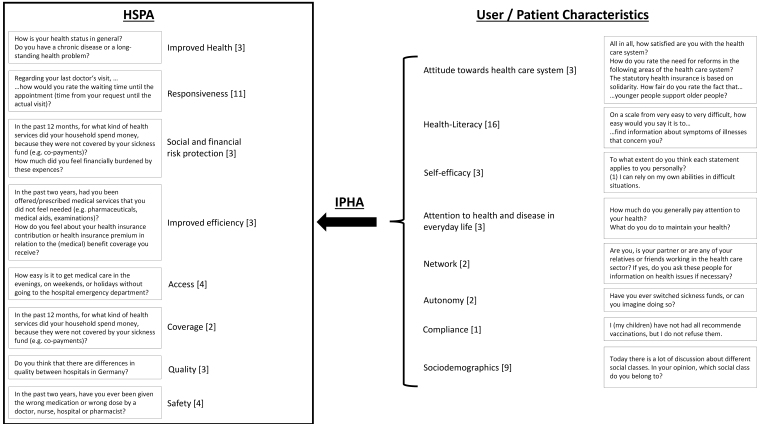
Survey instrument: Topics, Examples for Questions and Number of Items. Abbreviations: HSPA, Health system performance assessment; IPHA, Health System Performance Assessment.

In addition, the concepts and results of a previous qualitative study (see *User characteristics*), are operationalised in 39 of the survey items for participants with SHI and 38 of the survey items for participants with PHI. Figure 2 presents the topics of the questionnaire with examples of the related items and the number of items.

Overall, 59 items have been validated and tested in various national and international studies. If necessary, only minor linguistic changes were made to these. The further 13 items have been developed especially for the research question of this study and a validation is still pending. However, pretests have already shown that the questions were clearly worded and understood.

#### Final Goals

*Improved Health:* This is measured using 3 items commonly used in national and international health surveys to assess self-reported health.^22,23^ The items are (1) “How is your health status in general?” with 5 answer categories ranging from “very good” to “very bad,” (2) “Do you have a chronic disease or a long-standing health problem?” with the answer categories “Yes, one,” “Yes, several” or “No,” and (3) a vertical visual analoguescale with which respondents can report their perceived health status on that day with a grade ranging from 0 (the worst possible health status) to 100 (the best possible health status), as is done in the EQ-5D.

*Responsiveness:* This dimension is operationalised in 11 items in total. The perceived responsiveness of the health system is measured with nine items. Seven of these are used in the short version of the Multi-Country Survey Study on Health and Responsiveness and cover 6 dimensions of responsiveness developed by the WHO (dignity, confidentiality, autonomy, communication, prompt attention, and choice of care provider).^24-26^ Two dimensions (social support and quality of basic amenities) are left out because a previous study on responsiveness found out that these dimensions are of minor relevance for the German healthcare sector. In their place, 2 dimensions that are of greater relevance for the German context were developed: coordination of care and trust, each operationalised by one item.^27^ The questions refer to the last physician consultation and the 5 answer categories range between “very good” and “very bad.”

In addition, the item “perceived discrimination” is used, which is part of health system responsiveness and an indicator of equity.^28^ Participants are asked if they have experienced any kind of discrimination related to their interaction with the healthcare system within the past 12 months. In 2 subsequent questions, participants can specify the area in which they perceived discrimination (eg, waiting times) and the reason(s) they thought they were disadvantaged (eg, age or religion).

To assess responsiveness beyond their interaction with healthcare providers, participants are also asked about their satisfaction with different aspects of the health system, eg, quality of care, level of out-of-pocket payments, availability of different healthcare providers, and coordination between providers.^29^ Satisfaction is operationalised as the perceived need for reform, and participants can choose between the answer categories “no need for reform,” “low need for reform,” “high need for reform,” and “I don’t know.”

*Social and financial protection:* The dimension of financial protection is operationalised in 3 items, one of which is also listed under the coverage dimension. To assess perceived financial burden, participants are asked how burdened they feel financially by certain expenses.^30^ The answer categories are “very strong,” “strong,” “fair,” “less strong,” and “not at all.” The questions on financial protection conclude with an item asking whether participants have ever had difficulty paying their health insurance contribution (for people with SHI) or health insurance premium (for people with PHI).

*Improved efficiency:* The multiple aspects of efficiency in healthcare make it difficult to define clear indicators. Potential waste, inefficient care and poor coordination are relevant issues.^10,31^ In this study, efficiency is operationalised by asking participants the following 2 questions: *“* In the past 2 years, did a test have to be ordered that had already been done due to a lack of coordination between ambulatory care doctors and/or hospitals?” and “In the past 2 years, were you offered/prescribed medical services that you felt you didn’t need (eg, pharmaceuticals, medical aids, examinations)?” In addition to reporting experiences with inefficient care, one additional item was developed to capture the perceived efficiency of the healthcare system: “How do you feel about your health insurance contribution or health insurance premium in relation to the (medical) coverage you receive? Is it…” with the answer categories “too high,” “high,” “fair,” “low” or “too low.”

#### Intermediate Goals

*Access:* Access is operationalised by the question “How easy is it to get medical care in the evenings, on weekends, or on holidays without going to the hospital emergency department?” with 4 answer categories ranging from “very easy” to “very difficult.”^32^

The indicator “unmet healthcare need” has become the dominant measure of access internationally.^33^ In the present study, it is operationalised by asking participants if they have experienced any situation within the past twelve months in which they did not seek healthcare because of (1) waiting time, (2) distance or problems with transportation, or (3) financial reasons. To assess the level of unmet need due to financial reasons in more detail, participants are given a list of healthcare services (eg, medical aids, dental treatment) to specify the kind of care they did not seek.

*Coverage:* Population coverage is not explicitly surveyed in the present study because the study sample consisted exclusively of people with SHI or PHI. Two other dimensions of health coverage, however, are the scope and scale of benefits. These are operationalised by providing participants with a list of health services that may not be, or are only partly, covered by their sickness fund or PHI. Participants are asked, “In the past 12 months, what kind of health services did your household spend money on because they were not covered by your sickness fund (eg, co-payments)?” The subsequent question refers to the level of out-of-pocket spending on non-covered healthcare services. Eight answer categories are given ranging from “up to 49 euros” to “3000 euros or more.”

*Quality:* Quality is measured by asking participants how important different hospital characteristics are and if they know and make use of public hospital reporting systems. The first question is as follows: “Suppose you have to go to the hospital for a simple and scheduled surgical procedure (eg, for hernia repair or a minor knee injury). Several hospitals are eligible and you have detailed information about these hospitals. Which 2 characteristics would be particularly important for you to choose between them?” This question is followed by the question “Do you think that there are differences in quality between hospitals in Germany?” Finally, participants are asked which sources of information about hospital quality they know, and whether they have already used them.

*Safety:* Based on the questionnaire of the Commonwealth Fund’s International Health Policy Survey,^34^ safety is measured in the present study using 4 items asking (1) if the participants have ever been given the wrong medication or wrong dose by a doctor, nurse, hospital or pharmacist in the past 2 years; (2) if there was a time the participants thought a medical mistake was made in their treatment or care in the past 2 years; (3) if the doctor or other health professional involved told the participants that a medical error had been made in their treatment in the past 2 years; and (4) if the participants have ever been given wrong results of medical or laboratory tests in the past 2 years.

#### User Characteristics

It can be assumed that undertaking an HSPA from a population perspective requires taking the individual characteristics of the system’s users into account. Usually, sociodemographic attributes and regional variations are used as explanatory variables to predict differences in dependent variables. In this study, the explanatory model is to be extended by including additional approaches to describe the system’s users. In the run-up to this study, qualitative interviews with 27 persons were conducted to explore in terms of which characteristics do individuals differ in their interaction with the health system and what are their underlying patterns of behaviour and action? The results of the qualitative interviews suggested that users’ past experiences and attitudes towards the principles of the health system are important factors for explaining differences in users’ interaction with the health system. Furthermore, the concepts of health literacy and self-efficacy play an important role in this regard. Other relevant characteristics are the attention an individual pays to his or her health or disease, the availability of a personal network, users’ autonomy, and compliance.

*Attitudes towards the health system:* A user’s general attitude towards the health systems was operationalised using the initial question “All in all, how satisfied are you with the healthcare system?” with 5 answer categories ranging from “very satisfied” to “very unsatisfied.”^35^ To put more emphasis on users’ attitudes towards the specifics of the German health system participants are asked, *“* SHI is based on the principles of solidarity. How fair do you rate the fact that…healthier people support sicker people?…younger people support older people?…singles and childless people support families with children?…higher incomes support lower incomes?” with 4 answer categories “absolutely fair,” “mostly fair,” “mostly unfair,” “absolutely unfair.”^36^

In Germany, there are ongoing discussions about whether people with PHI receive favourable healthcare compared to people with SHI.^37^ In order to capture people’s views on this matter, the following question is included: “Do you feel that the healthcare of people with SHI differs from that of people with PHI?” Three answer categories are given: “no difference,” “healthcare is better for people with SHI” and “healthcare is better for people with PHI.”

*Health literacy:* Health literacy can be defined as the skills, knowledge and motivation to access, understand, assess and apply information to form an opinion and make decisions about healthcare, prevention and health promotion in everyday life that helps people maintain or improve their health and quality of life.^38^ HLS-EU-Q16 is used to measure the level of health literacy, which is an internationally applied instrument developed by the European Health Literacy Project.^39,40^

*Self-efficacy:* Self-efficacy describes the individual’s ability to cope successfully with difficulties in everyday life.^41^ The results of various studies suggest that self-efficacy is an important predictor of health behaviour and health beliefs.^42^ In the present study, the validated “General Self-Efficacy Short Scale” is used to measure self-efficacy. This scale contains 3 items and has been used in several surveys.^43^

*Attention to health and disease in everyday life:* The attention an individual pays to his or her health or disease is closely linked to issues of health behaviour. This area is introduced with the question “How much do you generally pay attention to your health?” with 5 answer categories from “very strong” to “not at all.” Subsequently, participants are asked what they do to maintain their health and are given 11 answer categories (eg, going for walks, taking vitamins, eating healthy, avoiding alcohol). With the third item, participants are asked if they have changed their health-related behaviour in the past 12 months and, if so, what their reasons for doing so have been.^44^

*Network* : The findings of the qualitative interviews undertaken before this study suggest that experiences with healthcare and personal health behaviour are strongly influenced by whether there are health professionals in the individual’s personal network. Therefore, the survey asks if the participants, their partners, or any relatives or friends work in the healthcare sector, and if the participants ever ask these people for information on health issues.

*Autonomy:* Autonomy is the capacity to make an informed and independent decision. The qualitative interviews undertaken in the run-up to the present study revealed that users of the health system differ in terms of their autonomy in interacting with healthcare providers, following health-related instructions, and interacting with the health system as a whole. The last of these points is operationalised by asking participants, “Have you ever switched sickness funds, or can you imagine doing so?” Participants who answer this question with “yes” are subsequently asked for the reasons for the (potential) switch. People with SHI have the right to choose their sickness fund freely.

*Compliance:* To measure the participants’ compliance in terms of actions they take in relation to their health, they are asked 2 questions about their attitude towards vaccinations for themselves and their children, if they have any. Participants can decide between the answers “I (my children) usually take recommended vaccinations,” “I (my children) have not had all recommended vaccinations, but I do not refuse them,” “I deliberately reject some vaccinations (for my children),” and “I generally reject vaccinations (for my children).”^45^

*Sociodemographic characteristics:* The respondents’ sociodemographic characteristics are captured using nine items. The 8 items gender, year of birth, total number of people in household, number of people in household who are <14 years, employment status, highest educational attainment, highest training qualification, and monthly net income of household allow an objective assessment of socio-economic status. In addition, one question was included to assess the subjective socio-economic status, as this has been hypothesised to influence how users assess health system performance. Subjective socio-economic status is operationalised with the item “Today there is a lot of discussion about different social classes. In your opinion, which social class do you belong to?” with 6 answer categories from “lower class” to “upper class” and “none of these.”

### Claims Data

To explore associations between insured characteristics and differences in the assessment of health system performance claims data on morbidity, care delivery, service utilisation, financial protection and sociodemographics are collected.

Claims data consists of the billing codes that healthcare providers (or patients) submit to payers and gives a holistic view of the patient’s interactions with the healthcare system. Claims data is used as the second data source in this study and is provided by a large regional sickness fund (AOK Nordost) and a PHI company operating nationwide (Debeka). The data covers 6 quarters, from January 1, 2017 to June 30, 2018, and reflects the period previous to the start of the survey in October 2018.

Table shows the topic related variables provided by SHI and PHI claims data. It is important to note that differences in financing mechanisms between statutory sickness funds and PHI companies lead to differences in the validity of claims data. In the statutory system, the healthcare providers bill the services provided directly with the respective sickness fund, so that every interaction is recorded in the accounting system of the sickness fund. On the other hand, privately insured persons pay in advance and submit the bills to their health insurance company for reimbursement. The amount of the deductible, as well as the time of the claim for reimbursement, may result in the services provided not being deposited in the health insurance billing system.

**Table:** Claims Data Record

**Topic**	**Variable**	**Indicator SHI**	**Indicator PHI**
Morbidity	Grade of long-term care entitlement	Grade 1/2/3 (out of 5)	Grade 1/2/3 (out of 5)
	Diagnoses in ambulatory care	ICD-10 codes	ICD-10 codes
	Diagnoses in hospital care	ICD-10 codes with discharge	ICD-10 codes with discharge
	Pharmaceutical care	ATC codes	ATC code, if pharmaceutical was prescribed for three consecutive quarters during reported period
Care delivery	Enrolment in DMP	Indication of DMP	n.a.
	Enrolment in ICM	Indication of ICM	n.a.
	Care Model	n.a.	Indication of Care Model
	Number of different physicians in ambulatory care	Practice ID of physician	Practice ID of physician
Service utilisation	Use of complementary medicine	ATC V60	ATC V60
	Number and length of hospital stays	Date of admission/ Date of discharge	Date of admission/date of discharge
	LTC expenditures	Expenditures in Euro (€)	Expenditures in Euro (€)
	SHI health expenditures:	Expenditures in Euro (€)	Expenditures in Euro (€)
	• ambulatory care		
	• pharmaceuticals		
	• hospitals		
	• medical aids		
	• healthcare professions		
	• home (ambulatory) nursing care		
	• rehabilitation transport		
Financial protection	Co-payment exemption	1%/2%	n.a.
	Co-payment to:	Co-payment in Euro (€)	n.a.
	• long-term care		
	• pharmaceuticals		
	• medical aids		
	• healthcare professions		
Sociodemographics	Age	Year of birth	Year of birth
	Gender	M/F	M/F
	Nationality	Nationality	n.a.
	Living area	Administrative county key/state	Administrative county key/state
	Insurance status	Mandatory members, dependents of mandatory members, pensioners, dependents of pensioners, voluntary members, dependents of voluntary members	n.a.
	Eligibility to aid allowances	n.a.	Yes/no
	Health insurance tariff	n.a.	Level of deductibles/basic tariff/standard tariff

Abbreviations: SHI, statutory health insurance; PHI, private health insurance; M/F, male/female; LTC, long-term care; ATC, anatomical therapeutic chemical; ICD, International Classification of Diseases; DMP, Disease Management Program; ID, identification number; ICM, Integrated Care Model; . Note: n.a. indicates that information is not applicable or available.

*Morbidity:* While the questionnaire records the self-reported health status, claims data allow an objective assessment of the health status based on documented medical diagnoses according to the International Classification of Diseases (ICD-10) coding in ambulatory and hospital care. Pharmaceutical care is another variable to estimate morbidity. While SHI claims data provide ATC (anatomical therapeutic chemical) codes of all prescribed pharmaceuticals in ambulatory care, PHI claims data provide ATC codes of pharmaceuticals that were prescribed for at least 3 consecutive quarters during the reported period. Based on this variable, it can be concluded that the insured has a chronic disease. Morbidity is further measured by the grade of long-term care. The higher the long-term care grade is, the larger is the amount of required help to carry out daily activities of everyday life due to a physical, psychological, mental disease, or handicap. Persons with a long-term care grade 4 or 5 are excluded from the original sample, since it can be assumed that completing the questionnaire is a much too great burden for this group of people.

*Care delivery:* Disease Management Program (DMP) and Integrated Care Model (ICM) have been introduced in the SHI system to improve coordination and quality of care for chronically ill people. In addition to improving health and stopping the progress of a disease, DMP and ICM also help improving the individual quality of life of the chronically ill. Similar to SHI-operated DMP and ICM, PHI companies offer care models for their insured persons.

*Service utilisation :* It is hypothesised that the volume of service utilisation affects the individual assessment of health system performance.^46^ SHI claims data are suitable for mapping service utilisation. The data is nearly complete, since all services billed to the sickness fund are recorded. The data of PHI, however, is not available to the same extent. Due to the differences in financing described above, only those services are recorded for which the insured has filed a claim for reimbursement. The actual utilisation is therefore probably higher. Service utilisation is operationalised by number and length of hospital stays, expenditures for long-term care, and SHI and PHI expenditures for health services by provider.

*Financial protection:* Financial protection is a final goal in the HSPA framework by WHO. In addition to the self-reported primary data on the amount of co-payments, claims data is used to provide information on the actual mandatory co-payments. Information on mandatory co-payments and the exemption of these are only available for SHI insured.

*Sociodemographics :* Claims data includes the variables age, gender, nationality and living area to describe the insured’s sociodemography. Claims data by PHI provides information about the eligibility to aid allowances and the chosen tariff.

### Course of Survey and Data Linkage

**Figure 3:**
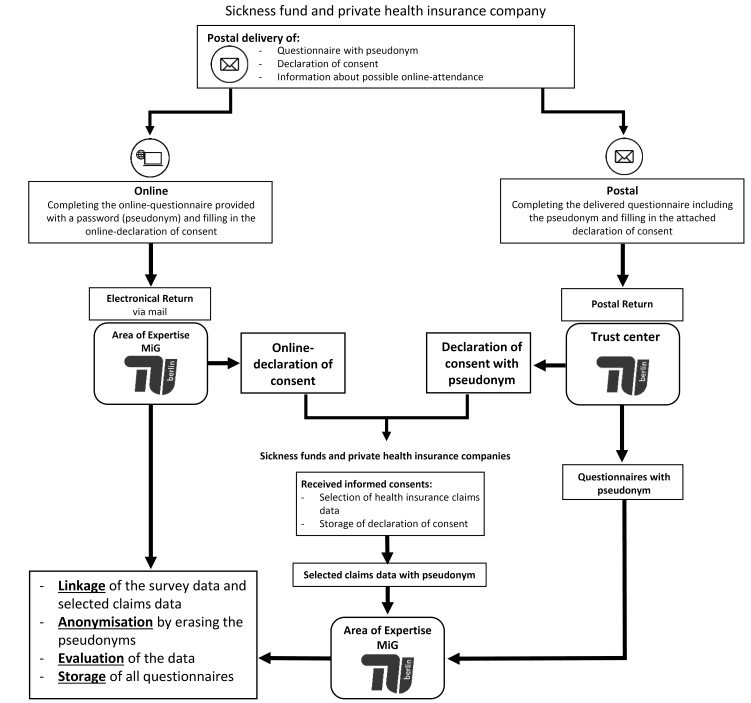
Linkage of Survey and Administrative Claims Data.

For each insured of the sample, a pseudonym is created. Both the questionnaire and the declaration of consent are provided with the pseudonym. The pseudonym serves as the key variable for linking the survey data to the claims data. Survey data and claims data are only linked and included in further analyses if the insured has signed and sent along the consent form or has given the consent to link the data online. To ensure that the researchers at Berlin University of Technology never have access to personal data from the consent form a trust centre is implemented. As shown in Figure 3 the trust centre is responsible for collecting the reply envelopes, splitting the questionnaires from the informed consents, and sending the questionnaires to Berlin University of Technology and the informed consents to the sickness fund and the private insurance company, respectively. After the data linkage has been completed, the pseudonym will be deleted, so that an anonymous data record is available for the following analyses.

### Planned Analysis

The final dataset does only include cases that filled in a minimum of 50% of the questionnaire and that are linked with administrative claims data. Preliminary analyses of the linked dataset include descriptive work to identify out-of-range values for variables, conflicting results, and possible data entry errors. Multiple imputation will be used for handling missing values. Descriptive statistics are used to present the population’s assessment of the German health system. Associations between HSPA outcomes and user characteristics are modelled in multivariate analyses. All analyses are conducted separately for SHI and PHI data.

## Discussion

So far, different elements of HSPA have already been subject to international research and published literature. Integrating the users’ interests, expectations, and perspectives into every level of the health system is highly relevant for sustainable and high performing health systems. The attempt to include the population perspective systematically within the performance assessment of the German health system is one of the major strengths and innovations of the present IPHA study. In addition, the study analyses differences in HSPA based on the identification of user types which vary in their morbidity und utilisation behaviour as well as in their interaction with the health system.

Therefore, a survey among statutorily and privately insured people in Germany is conducted. Survey data is linked with claims data if informed consent has been given by survey participants. This study protocol demonstrates the development of the study design including a detailed description of the survey instrument and the claims data record as well as the procedure to link these datasets.

Whereas research based on health insurance claims data has already been done for several decades in Germany, the linkage of survey data with claims data at an individual level is a relatively new field of research. In particular, the use of PHI data is so far rather rare. On the one hand this creates new opportunities for analyses in the field of health services research, on the other hand the associated regulations of data protection as well as the heterogeneity of the German health insurances make this approach difficult and implicate some limitations.

A random sample of both SHI-insured and PHI-insured is drawn in order to be as representative as possible for the German population. However, it has to be considered that AOK Nordost is a regional sickness fund with a structure of insured people who do not fully represent the German SHI-insured population. For example, AOK Nordost insured people have on average a higher age and more often a foreign nationality. In order to avoid a bias, the sample is stratified by age and younger age groups are overrepresented by 10%. The nationality of SHI-insured is recorded in the claims data and will be included as a control variable in the analyses. The completion of the questionnaire with its 71 items requires a high willingness for participation. Combined with signing the informed consent for data linkage, the study participation is a rather high effort for the survey participants. Since both parts are necessary for fulfilling the inclusion criteria, it is very likely that the response rate is affected. Therefore, the sample size of PHI-insured is also overrepresented by 10% especially in younger working-age groups, who tend to have less spare time.

The high standard of the questionnaire is also the reason why people who have a long-term care degree of 4 or 5 and severely limited everyday abilities and those who receive end-of-life care have been excluded from the sample. This group represents a large part of the population with a high utilization of healthcare. It is of great importance in HSPA, which is why future research should also use/develop appropriate methods to refer to this population group.

In the development of the questionnaire some limitations of the population perspective in HSPA in a cross-sectional survey became evident. Overall the whole questionnaire is rather cognitive demanding, although it was tried to phrase questions as simple as possible. Yet, this will be part of the analysis: are there questions with many missing values, are certain characteristics of respondents associated with not answering questions, do responder and non-responder differ systematically in the available information based on claims data?

Furthermore, some concepts are difficult to assess in a cross-sectional survey. While assessing “improved health” might sound straightforward at first sight, it has to be considered, that ideally the improved health only related to health improved by healthcare actions (prevention, medical care, etc). It also makes a distinction necessary between the population health that goes into the system and can be addressed by healthcare (the need of the population) and the population health as a result of healthcare. Yet, it was for us not possible to address these differentiations with the survey. We therefore decided to use standardized questions to assess population health and analyse, among others, if these reflect the “objective” health status as it is documented within the claims data. Ideally in future longitudinal studies should be conducted in order to fully address the issue of “improved health.”

PHI claims data has hardly been used in German healthcare service research so far. And to our knowledge this is the first time that PHI claims data is linked with survey data. In addition to the already mentioned advantages of this approach, however, the PHI claims data have some peculiarities that have to be considered in the interpretation of the results. Due to the reimbursement principle in PHI, in which insured persons pay their service provider directly and then get reimbursed by their insurance, the actual utilization of health services might not be registered with the insurance company. In addition, the insured can submit bills very late, so that the treatment may not have taken place during the observation period. Any resulting under- or overestimation of healthcare utilization will be taken into account in the analysis of the data.

Despite some limitations, the linkage of different data sources is of high potential for assessing and analysing determinants of health system performance from the population perspective. Specific patterns of interaction and use of the health system can be identified and linked with users’ expectations and opinions. The results from the IPHA study provide new insights in the population perspective of HSPA. As a result, user-oriented implications and improvements of the health system can be identified and provided to German health system decision-makers. The methodology applied can also be used to integrate the population perspective into studying other health systems.

## Acknowledgements

The study IPHA is part of the Berlin Centre for Health Economics Research (BerlinHECOR), Berlin, Germany and receives funding from the German Federal Ministry of Education and Research under grant agreement n° 01EH1604A.

## Ethical issues

The study protocol was approved by the ethics committee of the Charité – Universitätsmedizin Berlin, Berlin, Germany. The project partners, in accordance with the respective data security engineers, have developed strict criteria (included in the project proposal) regarding the sampling of patients, the mailing of the questionnaire, and the storage, linkage and access of the data to secure the privacy and confidentiality of the data at all times during the project.

## Competing interests

Authors declare that they have no competing interests.

## Authors’ contributions

MB and JR had the main role in manuscript preparation, supported by JK and KA. RB, JR, and MB had the main responsibility for developing the research question and the study design. MB, JR, JK, and KA developed the questionnaire and the claims data query. JK and MB have the main role in preparing the postal and online survey. JR, MB, JK, and KA will be responsible for the upcoming data analyses. All authors were responsible for reading, commenting upon, and approving the final manuscript.

## References

[1] World Health Organization Regional Office for Europe. Pathways to health system performance assessment: a manual to conducting health system performance assessment at national or subnational level. Copenhagen: WHO Regional Office for Europe; 2013.

[2] Nuti S, Vola F, Bonini A, Vainieri M (2016). Making governance work in the health care sector: evidence from a ‘natural experiment’ in Italy. Health Econ Policy Law.

[3] Organisation for Economic Co-operation and Development (OCED). Health at a Glance: Europe 2018. Paris: OECD; 2018.

[4] Tawfik-Shukor AR, Klazinga NS, Arah OA (2007). Comparing health system performance assessment and management approaches in the Netherlands and Ontario, Canada. BMC Health Serv Res.

[5] World Health Organization (WHO). Everybody’s business: strengthening health systems to improve health outcomes: WHO’s framework for action. Geneva: WHO; 2007.

[6] World Health Organization (WHO). The world health report 2000: health systems: improving performance. Geneva: WHO; 2000.

[7] World Health Organization (WHO). Monitoring the building blocks of health systems: a handbook of indicators and their measurement strategies. Geneva: WHO; 2010.

[8] Dorota BE. So what? Strategies across Europe to assess quality of care: report by the expert group on health systems performance assessment. Luxembourg: Publications Office of the European Union; 2016.

[9] Organisation for Economic Co-operation and Development (OCED). Health at a Glance 2017: OECD Indicators. Paris: OECD; 2017.

[10] Cylus J, Papanicolas I, Smith PC, eds. Health system efficiency: how to make measurement matter for policy and management. Copenhagen: WHO Regional Office for Europe; 2016. 28783269

[11] Valentine NB, Prasad A, Rice N, Robone S, Chatterji S. Health systems responsiveness: a measure of the acceptability of health-care processes and systems from the user’s perspective. In: Smith PC, Mossialos E, Papanicolas I, Leatherman S, eds. Performance measurement for health system improvement: experiences, challenges and prospects. 1st ed. Cambridge: Cambridge University Press; 2009:138-186.

[12] Busse R. Understanding satisfaction, responsiveness and experience with the health system. In: Papanicolas I, Smith PC, eds. Health system performance comparison: An agenda for policy, information and research. European Observatory on Health Systems and Policies series. Maidenhead, England: Open University Press; 2013:255-279.

[13] Coulter A, Cleary PD (2001). Patients’ experiences with hospital care in five countries. Health Aff (Millwood).

[14] Weldring T, Smith SM (2013). Patient-Reported Outcomes (PROs) and Patient-Reported Outcome Measures (PROMs). Health Serv Insights.

[15] Fujisawa R, Klazinga NS. Measuring patient experiences (PREMS): progress made by the OECD and its member countries between 2006 and 2016. OECD Health Working Papers. No. 102. Paris: OECD; 2017.

[16] Sirven N, Santos-Eggimann B, Spagnoli J (2012). Comparability of health care responsiveness in Europe. Soc Indic Res.

[17] Bleich SN, Ozaltin E, Murray CK (2009). How does satisfaction with the health-care system relate to patient experience?. Bull World Health Organ.

[18] Anzahl der Krankenkassen im Zeitverlauf- Konzentrationsprozess durch Fusionen (Angaben zum Stichtag 1.1). GKV-Spitzenverband; 2018.

[19] Versicherte je System in Prozent. GKV-Spitzenverband; 2019.

[20] Zahlen und Fakten. Verband der Privaten Krankenversicherung; 2018.

[21] Röttger J, Blümel M, Engel S (2015). Exploring health system responsiveness in ambulatory care and disease management and its relation to other dimensions of health system performance (RAC) - study design and methodology. Int J Health Policy Manag.

[22] Brooks R, Rabin R, de Charro F. The Measurement and Valuation of Health Status Using EQ-5D: A European Perspective: Evidence from the EuroQol BIOMED Research Programme. Dordrchet: Kluwer Academic; 2003.

[23] Statistische Ämter des Bundes und der Länder. Leben in Europa 2013 Personenfragebogen; 2013.

[24] Valentine N, Darby C, Bonsel GJ (2008). Which aspects of non-clinical quality of care are most important? Results from WHO’s general population surveys of “health systems responsiveness” in 41 countries. Soc Sci Med.

[25] Üstün TB, Sonmath C, Abdelhay M, Murray CJL, WHS Collaborating Group. The World Health Surveys. In: Murray CJL, Evans DB, eds. Health systems performance assessment: debates, methods and empiricism. Geneva: WHO; 2003.

[26] de Silva A. A framework for measuring responsiveness. GPE Discussion Paper Series: No. 22 32. Copenhagen: World Health Organization; 2000.

[27] Röttger J, Blümel M, Fuchs S, Busse R (2014). Assessing the responsiveness of chronic disease care - is the World Health Organization’s concept of health system responsiveness applicable?. Soc Sci Med.

[28] Alvarez-Galvez J, Salvador-Carulla L (2013). Perceived discrimination and self-rated health in Europe: evidence from the European Social Survey (2010). PLoS One.

[29] Böcken J, Braun B, Meierjürgen R. Gesundheitsmonitor 2016: Bürgerorientierung im Gesundheitswesen. Gütersloh: Verlag Bertelsmann Stiftung; 2016.

[30] Statistische Ämter des Bundes und der Länder. Leben in Europa 2017 Personenfragebogen; 2017.

[31] Organisation for Economic Co-operation and Development (OCED). Tackling Wasteful Spending on Health. Paris: OECD; 2017.

[32] Schoen C, Davis K, Stremikis K. Mirror, Mirror on the Wall: How the Performance of the U.S. Health Care System Compares Internationally, 2010 Update. The Commonwealth Fund; 2010.

[33] Perić N, Hofmarcher MM, Simon J (2018). Headline indicators for monitoring the performance of health systems: findings from the european Health Systems_Indicator (euHS_I) survey. Arch Public Health.

[34] Osborn R, Squires D, Doty MM, Sarnak DO, Schneider EC (2016). In new survey of eleven countries, US adults still struggle with access to and affordability of health care. Health Aff (Millwood).

[35] Böcken J, Braun B, Meierjürgen R. Gesundheitsmonitor 2014: Bürgerorientierung im Gesundheitswesen. Gütersloh: Verlag Bertelsmann Stiftung; 2014.

[36] Braun B, Böcken J, Landmann J. Gesundheitsmonitor 2010: Bürgerorientierung im Gesundheitswesen. Gütersloh: Verlag Bertelsmann Stiftung; 2011.

[37] Himmel K, Schneider U. Ambulatory Care at the End of a Billing Period. Research Paper 14. Hamburg Center for Health Economics; 2017.

[38] Sørensen K, Van den Broucke S, Fullam J (2012). Health literacy and public health: a systematic review and integration of definitions and models. BMC Public Health.

[39] The HLS-EU Consortium. HLS-EU-Q - Measurement of health literacy in Europe: HLS-EU-Q47, HLS-EU-Q16, and HLS-EU-Q 86; 2012.

[40] Sørensen K, Pelikan JM, Röthlin F (2015). Health literacy in Europe: comparative results of the European health literacy survey (HLS-EU). Eur J Public Health.

[41] Bandura A. Self-efficacy and health behaviour. In: Baum A, Newman S, Wienman J, West R, McManus C, ed. Cambridge Handbook of Psychology, Health and Medicine. Cambridge: Cambridge University Press; 1992:160-162.

[42] Glanz K, Rimer BK, Viswanath K. Health Behavior and Health Education: Theory, Research, and Practice. John Wiley & Sons; 2008.

[43] Beierlein C, Kovaleva A, Kemper CJ, Rammstedt B. Ein Messinstrument zur Erfassung subjektiver Kompetenzerwartungen: Allgemeine Selbstwirksamkeit Kurzskala (ASKU). GESIS-Working Papers 17. GESIS - Leibniz-Institut für Sozialwissenschaften; 2012.

[44] Altenhöner T, Philippi M, Böcken J (2014). Health behaviour and changes in health behaviour - are education and social status relevant? (German). Gesundheitswesen.

[45] Koch-Institut R (2017). Fragebogen zur Studie “Gesundheit in Deutschland aktuell”: GEDA 2014/2015-EHIS. Journal of Health Monitoring.

[46] Coulter A (2006). Can patients assess the quality of health care?. BMJ.

